# Metacognition as a transdiagnostic factor across eating disorders: a latent profile analysis study

**DOI:** 10.3389/fpsyg.2024.1391715

**Published:** 2024-06-24

**Authors:** Matteo Aloi, Antonino Carcione, Gianluca Lo Coco, Marianna Rania, Elvira Anna Carbone, Renato de Filippis, Cristina Segura-Garcia, Marco Tullio Liuzza

**Affiliations:** ^1^Department of Clinical and Experimental Medicine, University of Messina, Messina, Italy; ^2^Department of Health Sciences, University “Magna Graecia” of Catanzaro, Catanzaro, Italy; ^3^Third Centre of Cognitive Psychotherapy, Italian School of Cognitive Psychotherapy (SICC), Rome, Italy; ^4^Department of Human Science, University “Guglielmo Marconi”, Rome, Italy; ^5^Department of Psychology, Educational Science and Human Movement, University of Palermo, Palermo, Italy; ^6^Outpatient Unit for Clinical Research and Treatment of Eating Disorders, University Hospital Renato Dulbecco, Catanzaro, Italy; ^7^Department of Medical and Surgical Sciences, University “Magna Graecia” of Catanzaro, Catanzaro, Italy

**Keywords:** eating disorders, metacognition, latent profile analysis, targeted intervention, childhood maltreatment, personality traits, emotional dysregulation, depression

## Abstract

**Background:**

Metacognition is a crucial aspect of understanding and attributing mental states, playing a key role in the psychopathology of eating disorders (EDs). This study aims to explore the diverse clinical profiles of metacognition among patients with EDs using latent profile analysis (LPA).

**Method:**

A total of 395 patients with a DSM-5 diagnosis of ED (116 AN-R, 30 AN/BP, 100 BN, 149 BED) participated in this study. They completed self-report measures assessing metacognition, eating psychopathology, depression, emotional dysregulation, personality traits, and childhood adversities. LPA and Welch ANOVAs were conducted to identify profiles based on metacognition scores and examine psychological differences between them. Logistic regression models were employed to explore associations between personal characteristics and different profiles.

**Results:**

A 3-class solution had a good fit to the data, revealing profiles of high functioning (HF), intermediate functioning (IF), and low functioning (LF) based on levels of metacognitive impairments. Participants in the IF group were older and had a higher BMI than those in the HF and LF groups. Individuals with BN were largely categorized into HF and LF profiles, whereas participants with BED were mainly included in the IF profile. Participants in the LF group reported an impaired psychological profile, with high levels of depression, emotional dysregulation, childhood adversity, and personality dysfunction. Multinomial logistic regression analyses showed significant associations between metacognitive profiles and emotional and neglect abuse, emotion dysregulation, and detachment.

**Conclusion:**

This exploratory study unveils distinct metacognitive profiles in EDs, providing a foundation for future research and targeted interventions. In this light, metacognitive interpersonal therapy could be a valid and effective treatment for EDs, as suggested by the initial promising results for these patients.

## 1 Introduction

Metacognition is a psychological construct that significantly contributes to recognizing and attributing mental states to oneself and others. It involves reflecting and analyzing mental states, ultimately utilizing this awareness to navigate interpersonal conflicts (Semerari et al., 2003). According to this framework, metacognition consists of five distinct sub-functions that interact and can be compromised individually: monitoring, integration, differentiation, understanding others’ mind/decentration, and mastery.

Monitoring involves identifying and defining our mental states, encompassing thoughts, emotions, desires, and motivations. Integration pertains to the general ability to reflect on various mental states, identifying internal conflicts and contradictions. Poor differentiation prevents individuals from maintaining a critical distance from their subjective mental representations. Understanding others’ minds refers to the ability to formulate plausible hypotheses about their mental states, and decentration is the use of that ability to adopt a non-egocentric perspective. Lastly, mastery involves employing psychological information about mental states to address problems of escalating complexity and is connected to activities focused on regulation and control (Semerari et al., 2007, 2012; Carcione et al., 2011).

Researchers and clinicians have theoretically suggested the presence of specific metacognitive failures in patients with eating disorders (EDs) (Olstad et al., 2015; Simonsen et al., 2020). Eating disorders are challenging to treat, and there is still a need to identify psychological mechanisms associated with disordered eating patterns that may serve as important targets for therapeutic interventions. The capacity to monitor, interpret, and regulate mental states is a crucial factor influencing emotion regulation processes related to eating behaviors (Vann et al., 2014; Leppanen et al., 2022). Moreover, research has shown that individuals with adverse childhood experiences are at increased risk for developing emotion dysregulation and eating problems (Michopoulos et al., 2015). While emotion dysregulation has been identified as a mediator between childhood trauma and eating psychopathology (Moulton et al., 2015), it remains unclear whether various patterns of metacognitive functioning are associated with emotional dysregulation and childhood trauma experiences in patients with EDs (Martin and Strodl, 2023).

It is noteworthy that the few existing studies on metacognition in patients with EDs mainly focused on individuals with anorexia nervosa (AN) and bulimia nervosa (BN) (Rothschild-Yakar et al., 2018, 2019; Sacchetti et al., 2019; Kjaersdam Telléus et al., 2023), whereas patients with binge eating disorder (BED) received little attention from researchers (Simonsen et al., 2020). Recently, two studies have investigated the role of metacognition in the severity of binge eating and as a possible treatment target in this population. The first study supported the idea that low self-monitoring metacognition and high negative urgency lead to a worsening of binge severity through the mediation of emotional dysregulation (Aloi et al., 2020); a network analysis study, on the other hand, reported that impaired self-monitoring metacognition and difficulties in impulse control were central nodes in the psychopathological network of BED, while eating symptoms appearing marginal (Aloi et al., 2021).

Although the link between metacognition and EDs has received some empirical support, there is still a need for a greater understanding of heterogeneity in their presentations. In this framework, latent profile analysis (LPA) may represent a suitable approach to better identify and describe the profile of individuals characterized by specific patterns of metacognition (Gibson, 1959; Lazarsfeld and Henry, 1968). LPA serves as a useful technique for conducting person-centered analyses, distinct from variable-centered approaches. Specifically, LPA is a statistical method involving continuous variables (i.e., indicators) to identify hidden subgroups within a population (i.e., latent profiles) using specific variables. This approach operates on the assumption that individuals can be categorized with differing probabilities into various profiles or groups (Williams and Kibowski, 2016).

To the best of our knowledge, LPA was been employed in only a study with patients with EDs to investigate metacognition patterns (Gagliardini et al., 2020). However, the authors used the Mentalization Imbalances Scale (MIS) (Gagliardini et al., 2018), a measure of mentalizing imbalances based on six subscales according to the model proposed by Fonagy and colleagues (Bateman and Fonagy, 2004, 2016; Luyten et al., 2012). In this study, four different profiles of impairments in the dimension of mentalizing were identified. These profiles were heterogeneous in EDs represented in each group and showed notable distinctions across multiple factors such as attachment style, emotion dysregulation, empathy, interpersonal reactivity, and reflective function (Gagliardini et al., 2020).

Metacognition and mentalization are two constructs with some overlap, both referring to the ability of human beings to reflect and reason about their own and others’ mental states, and both attributing a multidimensional nature to these functions (Semerari et al., 2003; Bateman and Fonagy, 2016). Some differences emerge in the definition of metacognition, which includes the ability to use psychological information to cope with distress and interpersonal problems (i.e., metacognitive mastery), with dysfunctions in this area correlated with specific psychopathology profiles (Carcione et al., 2011). Furthermore, unlike mentalization, metacognition does not presuppose activation of the attachment system as the main source of disorders (Dimaggio et al., 2007). In this framework, the DSM-5 emphasizes the importance of reflective abilities. According to the alternative model of personality disorder (AMPD) of Section III, diagnosing a personality disorder requires assessing an individual’s personality functioning, which includes their ability to (1) self-reflect, thereby fostering a stable sense of self and self-direction, and (2) understand others’ perspectives to establish and maintain empathetic and healthy relationships (American Psychiatric Association, 2013).

Building on these previous findings, the present study specifically investigates the role of metacognition according to the model proposed by Semerari and colleagues (Semerari et al., 2007; Carcione et al., 2021). This model suggests that the measurement of this construct includes different and relatively independent sub-functions that could be impaired in several psychiatric disorders (Semerari et al., 2015; Carcione et al., 2019; Aloi et al., 2023).

The primary objective of this study was to explore the metacognition profiles that can be detected in patients with EDs, based on the metacognition sub-functions. The secondary aim was to investigate how these empirically derived metacognitive profiles are associated with some clinical, personality, and ED-related factors. Specifically, we examined whether patients belonging to the different metacognition profiles differ in the levels of childhood maltreatment (CM), negative affectivity, and emotion dysregulation, given the previous evidence that supported their interplay with metacognition and dysfunctional eating (Moulton et al., 2015; Martin and Strodl, 2023). Finally, we investigated which psychological characteristics are associated with the different profiles. Due to the exploratory nature of the present study and the limited existing literature, no specific hypotheses about these metacognitive profiles were formulated.

## 2 Methods

### 2.1 Participants

Participants eligible for inclusion were selected among those seeking care in the Outpatient Unit for Clinical Research and Treatment of Eating Disorders at the University Hospital “Renato Dulbecco” of Catanzaro (Italy) between June 2018 and June 2023. They were consecutively recruited during their initial visit for participation in this cross-sectional study and the aim and the description of the research were presented by the research team. To be included, patients needed to be aged between 14 and 65, diagnosed with an ED according to DSM-5 criteria (American Psychiatric Association, 2013), willing to take part, and able to provide valid informed consent. Exclusion criteria included comorbidity with severe psychiatric diagnoses (e.g., neurodevelopmental, schizophrenia spectrum, bipolar disorders, neurocognitive disorders), neurological or medical conditions (e.g., diabetes), active substance dependence or abuse (within ≤6 months), and other medical comorbidities or treatments that could influence eating behaviors.

Each participant underwent a diagnostic interview conducted by experienced psychiatrists through the Structured Clinical Interview for the DSM-5 (SCID-5-CV) (First, 2016) and the Eating Disorder Examination (EDE 17.0D) (Calugi et al., 2015). Afterward, participants were asked to complete self-report questionnaires aimed at assessing psychological aspects such as metacognition, depression, emotional dysregulation, childhood trauma, and personality traits.

Out of the 413 patients initially approached for the study, 18 were excluded during the screening or enrollment phase for the following reasons: five patients (1.2%) were not eligible due to active substance use disorder; five (1.2%) dropped out before the end of the assessment and were thus excluded from the study; four (1.0%) met the exclusion criteria for intellectual disability, and four (1.0%) were not eligible due to psychotic symptoms. Consequently, the final sample consisted of 395 patients (*N* = 116 AN-R, *N* = 30 AN/BP, *N* = 100 BN, *N* = 149 BED) with a dropout rate of 4.4%.

Only the patients who accepted to participate in the research protocol, provided informed consent, and completed the evaluation were included in the analysis. No missing data were reported in the participants’ socio-demographic information or in the assessment. This study adhered to the ethical principles outlined in the updated Helsinki Declaration (World Medical Association, 2013) and received approval from the Ethical Committee of “Regione Calabria, Sezione Area Centro” (identifier: Prot. 66/15.03.2018). Before completing the questionnaires, participants provided written informed consent. For minors, consent was acquired from their parents or legal tutors after providing detailed information.

### 2.2 Measures

- Eating Disorder Examination (EDE): This clinical interview assesses eating psychopathology’s presence and severity across four subscales using 28 items: Eating Restraint, Eating Concern, Weight Concern, and Shape Concern, contributing to a global EDE score (Calugi et al., 2015). This semi-structured interview delves into ED-related behaviors and psychopathology within the preceding 3 months. It examines behavioral symptoms associated with EDs, such as binge eating, self-induced vomiting, diuretic and laxative misuse, excessive exercise, and food restriction. Elevated scores signify a heightened severity of psychopathology. For this study, we only took into consideration the EDE total score; the total McDonald’s ω was 0.88.
- Beck Depression Inventory-II (BDI-II): This instrument measures the severity of depression through 21 items (Ghisi et al., 2006). Higher scores indicate more severe symptoms. Scores fall into these standardized categories: 0–13: minimal depression; 14–19: mild depression; 20–28: moderate depression; 29–63: severe depression. The reliability index, measured by McDonald’s ω, was 0.90.
- Metacognition Self-Assessment Scale (MSAS): This self-report questionnaire (Pedone et al., 2017) evaluates metacognitive functioning through 18 Likert-type response format items. Lower scores indicate impaired self-evaluation of metacognitive abilities across four domains: self-monitoring, critical distance (differentiation/decentration), mastery, and understanding others’ minds. McDonald’s ω internal consistency ranged from 0.83 (Mastery) to 0.92 (Self-monitoring) in this study.
- Difficulties in Emotion Regulation Scale (DERS): Comprising 36 items, the DERS (Giromini et al., 2012) evaluates emotion dysregulation across six subscales. The total score reflects overall problems in emotional regulation. In this study, the McDonald’s ω internal consistency coefficient for the total score was 0.88.
- Personality Inventory for DSM-5 (PID-5): This 220-item self-administered scale (Fossati et al., 2013) measures 25 personality traits grouped into five domains: negative affectivity, detachment, antagonism, disinhibition, and psychoticism. Participants rate items on a Likert scale. Higher scores are indicative of higher dysfunction in specific personality facets or domains. Internal consistency in this sample for the domains ranged from McDonald’s ω 0.84 (Negative affectivity) to 0.91 (Detachment).
- Childhood Trauma Questionnaire Short-Form (CTQ-SF): It is a self-administered test comprising 28 Likert-scale items measuring CM across five subscales (Innamorati et al., 2016). In the present study, internal consistency measured through McDonald’s omega ranged as follows: physical abuse 0.83; emotional abuse 0.89; sexual abuse 0.85; emotional neglect 0.92 and physical neglect 0.83.

### 2.3 Statistical analyses

Analyses were carried out with SPSS Version 26.0 and R Version 4.0.0 using the *tidyLPA* package (Rosenberg et al., 2018).

To establish the number of profiles within the sample, various models ranging from one to five profiles were assessed using information statistical criteria such as Consistent Akaike’s Information Criteria (cAIC), Bayesian Information Criteria (BIC), sample size-adjusted BIC (saBIC), and approximate weight of evidence criterion (AWE). Lower values of these indices indicate better predictive accuracy.

The accuracy of participant classification was evaluated using standardized entropy (ranging from 0 to 1), where values exceeding 0.80 suggest strong group differentiation (Ramaswamy et al., 1993).

Regarding sample size, there is no definitive recommendation for the minimum sample size in LPA. However, previous research suggests that a range of N ~ 300–1,000 tends to offer reliable fit indices for mixture models (Nylund-Gibson and Choi, 2018). The sample size in this study (*N* = 395) was considered adequate for the exploratory latent profile.

To empirically distinguish profiles linked to metacognition impairments in individuals with EDs, we conducted an LPA based on their scores across the four subscales of the MSAS.

Following the identification of an LPA solution, individuals were assigned to a class based on their most likely class membership. In case of departure from the homoscedasticity assumption, Welch ANOVA, followed by Dunnet T3 *post hoc* tests, was employed to identify differences among the empirically derived profiles in the self-reported measures. We also reported the eta-squared (η^2^), as a measure of the effect size of ANOVA.

Finally, scores were transformed into z-scores and a series of univariate multinomial logistic regressions were employed to explore relationships between profile memberships and the variables of interest (i.e., ED psychopathology, depression, childhood adversities, personality traits, emotion dysregulation).

Statistical significance was defined at *p* < 0.05.

## 3 Results

### 3.1 Latent profile analysis

**Figure 1:**
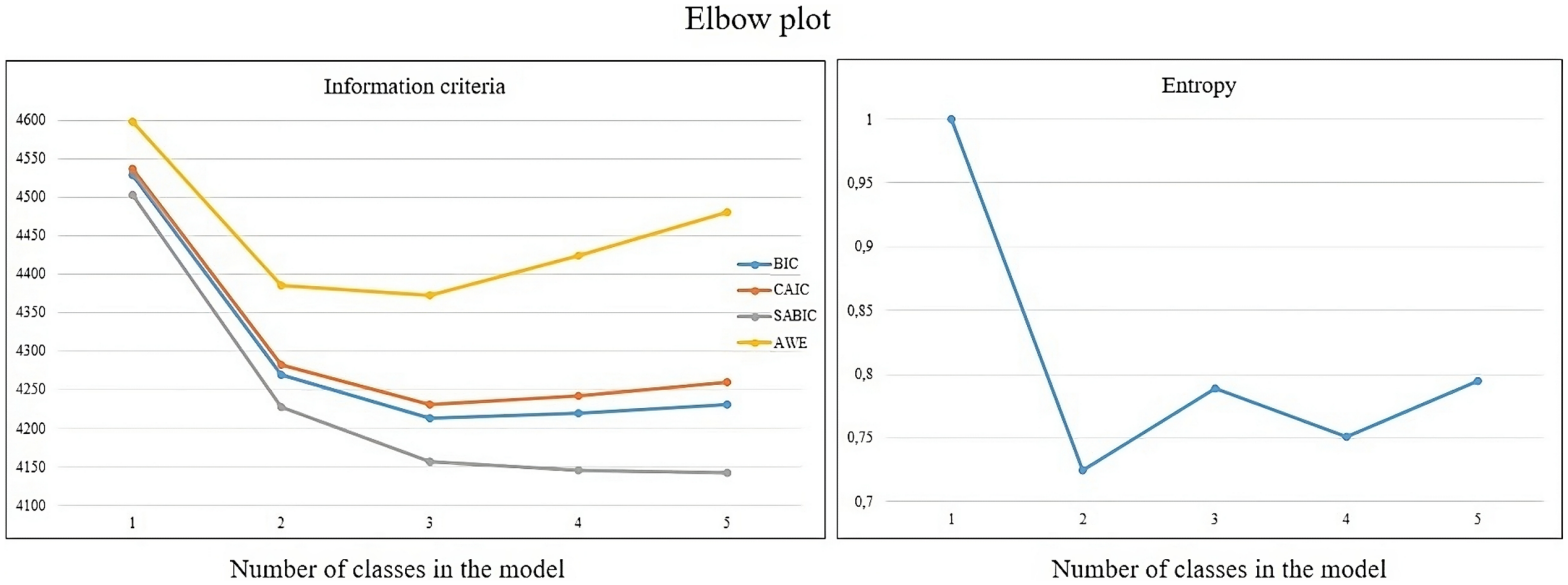
Fit indices for the latent profile analysis of the MSAS, with values of the information criteria (on the left), and the entropy (on the right).

The 3-class solution emerged as the most balanced option, considering the statistics used for evaluating model fit comparisons. Notably, the elbow plot showed a more pronounced change in information criteria between the 2-profile and 3-profile models (Figure 1). Overall, the three-class model seemed to fit the data best. This model exhibited the lowest AIC, cAIC, BIC, saBIC, and AWE values. Furthermore, the three-class model displayed the highest entropy (0.80), signifying a strong distinction between profiles (Ramaswamy et al., 1993).

**Figure 2:**
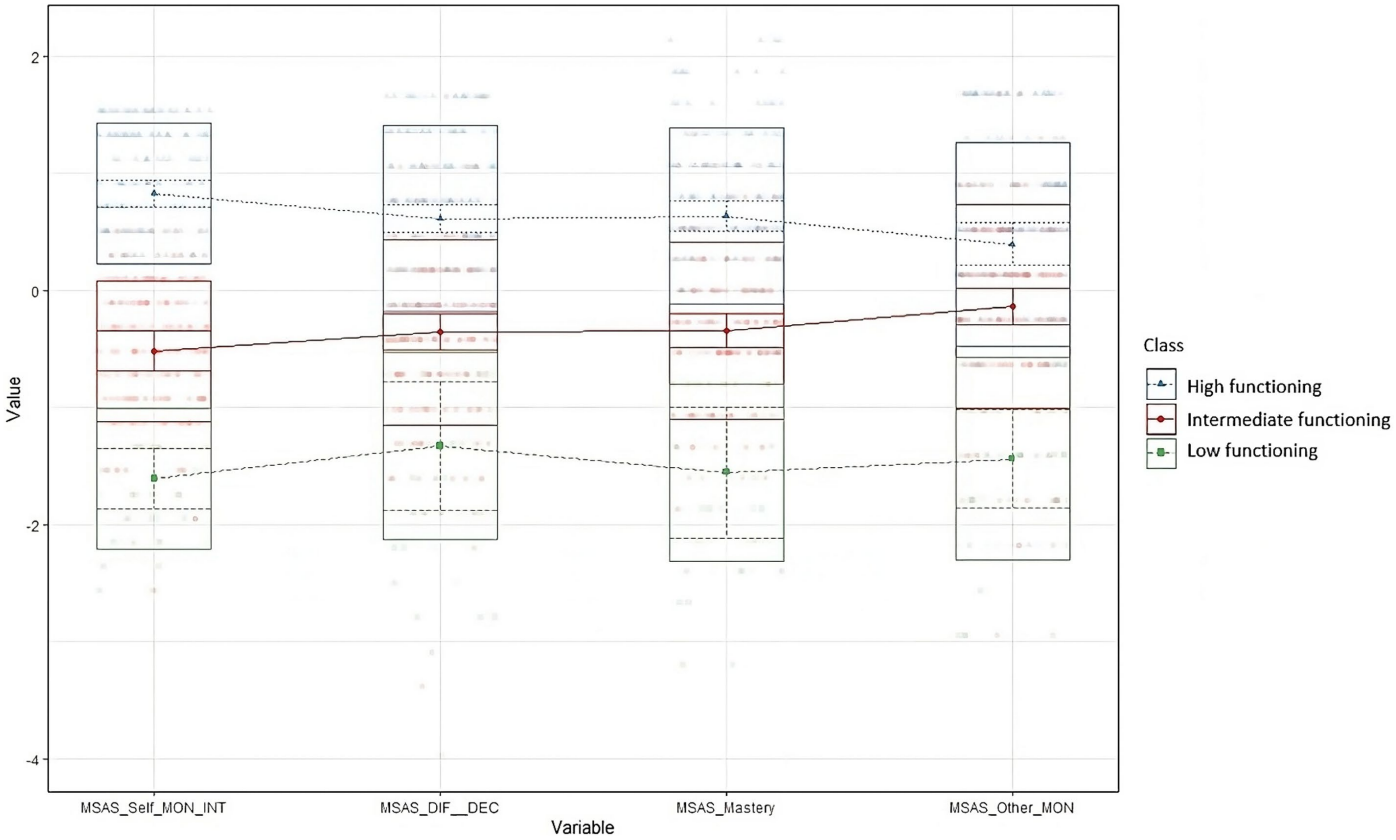
Standardized group averages on MSAS subscales for a three profile solution. MSAS, Metacognition Self Assessment Scale; Self_MON_INT, Self monitoring and integration; DIF_DEC, Differentiation/Decentration; Other_MON, Monitoring others’ cognitions.

This solution yielded a baseline class with high functioning (HF) according to the MSAS scores, including 191 (48.3%) participants. Members of this HF class reported high scores on each metacognition MSAS domain. A second class including 176 (44.5%) participants showed an intermediate functioning (IF) profile, characterized by moderate levels of understanding of others’ mind domain, which was close to those of the HF class. In contrast, the self-monitoring was different from the HF group. A third profile including 28 participants (7.2%) showed a low MSAS functioning (LF), characterized by the worst scores in MSAS domains. Figure 2 illustrates the standardized group averages on MSAS subscales for the three-profile solution.

Table 1 reassumes the characteristics of the total sample and each MSAS profile, including demographic features. Most participants were females, had completed middle and high school, and were single. Differences were found in age and BMI, with those in the IF profile reporting higher scores than those in the HF and LF profiles. Regarding BMI, the mean and SD for each diagnosis are as follows: AN-R (*M* = 17.5 ± 2.5 SD), AN-BP (*M* = 19.1 ± 2.5 SD), BN (*M* = 23.8 ± 5.3 SD) and BED (*M* = 38.8 ± 8.2 SD).

**Table 1:** Characteristics of the total sample and by latent profiles.

		Total sample	Low-functioning (LF)	Intermediate functioning (IF)	High-functioning (HF)			
		*N =* 395	*n =* 28	*n =* 176	*n =* 191	*χ*^2^/*F*	*p*	*Post hoc*
Age^a^		27.5 (13.6)	23.5 (13.2)	31.1 (14.3)	24.7 (12.3)	11.185	<0.001	IF > HF, LF
Sex	Female	366 (92.7)	27 (92.7)	164 (93.2)	175 (91.6)	0.957	0.620	
	Male	29 (7.3)	1 (3.6)	12 (6.8)	16 (8.4)			
Body mass index^a^		27.4 (11.1)	23.2 (6.6)	30.2 (12.5)	25.1 (9.3)	10.691	<0.001	IF > HF, LF
Civil status	Married	102 (25.8)	4 (14.3)	52 (29.5)	46 (24.1)	7.094	0.131	
	Single	283 (71.6)	24 (85.7)	117 (66.5)	142 (74.3)			
	Divorced	10 (2.5)	0 (0.0)	7 (4.0)	3 (1.6)			
Education	Elementary	6 (1.5)	2 (7.1)	1 (0.6)	3 (1.6)	34.847	<0.001	
	Middle school	170 (43.0)	15 (53.6)	55 (31.3)	100 (52.4)			
	High school	171 (43.3)	10 (35.7)	85 (48.3)	76 (39.8)			
	Master	48 (12.2)	1 (3.6)	35 (19.9)	12 (6.3)			

a Data are expressed as means and standard deviation.

Table 2 displays the distribution of participants within the three profiles. Based on the LPA analyses, individuals with BN mainly belonged to the LF and HF profiles. Nearly half of the participants with BED were included in the IF profile.

**Table 2:** The frequencies of eating disorder diagnosis across the three profiles.

	Low-functioning	Intermediate-functioning	High-functioning	Frequencies
AN-R	6 (21.4)	53 (30.1)	57 (29.8)	116
AN-BP	4 (14.3)	11 (6.3)	15 (7.9)	30
BN	11 (39.3)	25 (14.2)	64 (33.5)	100
BED	7 (25)	87 (49.4)	55 (28.8)	149
Total	28 (100)	176 (100)	191 (100)	395

Data are presented as frequencies (%) AN-R, Anorexia nervosa – restricting type; AN-BP, Anorexia nervosa – binge/purge type; BN, Bulimia nervosa; BED, Binge eating disorder.

### 3.2 Comparison of psychopathological variables between the three profiles

Table 3 shows the mean scores and comparisons between the three profiles on the EDE, BDI-II, DERS, PID-5, and CTQ-SF. Both the LF and IF profiles reported greater depressive symptoms and higher scores on four domains of PID-5 (i.e., negative affectivity, antagonism, disinhibition, and psychoticism) compared to the HF profile. The LF group reported the most severe scores on CM (i.e., emotional abuse and neglect, and physical neglect), emotion dysregulation, and the detachment domains of PID-5.

**Table 3:** Clinical characteristics of the total sample and by latent profiles.

		High-functioning (HF)		Intermediate-functioning (IF)		Low-functioning (LF)					
		Mean	SD	Mean	SD	Mean	SD	*F*	Sig.	ƞ^2^^a^	Dunnett T3
EDE	Total score	3.5	1.4	4.2	1.1	4.0	1.8	11.203	**<0.001**	0.06	IF > HF
BDI-II	Total score	23.8	12.5	31.8	13.2	38.0	11.8	17.935	**<0.001**	0.12	LF, IF > HF
DERS	Total score	91.2	26.0	119.9	25.0	142.0	27.8	74.710	**<0.001**	0.30	LF > IF, HF; IF > HF
PID-5	Negative affectivity	1.2	0.4	1.7	0.4	1.9	0.4	49.713	**<0.001**	0.23	LF, IF > HF
	Detachment	1.1	0.5	1.5	0.5	1.9	0.5	47.008	**<0.001**	0.23	LF > IF, HF; IF > HF
	Antagonism	0.7	0.4	0.9	0.5	1.1	0.5	16.412	**<0.001**	0.10	LF, IF > HF
	Disinhibition	0.9	0.4	1.3	0.4	1.6	0.5	40.874	**<0.001**	0.22	LF, IF > HF
	Psychoticism	0.7	0.5	1.2	0.6	1.5	0.7	37.284	**<0.001**	0.20	LF, IF > HF
CTQ-SF	Emotional abuse	8.9	4.0	9.7	4.8	13.3	5.7	7.045	**0.002**	0.06	LF > IF, HF
	Physical abuse	5.9	2.1	6.2	2.6	6.8	3.3	1.368	0.262		
	Sexual abuse	6.7	4.0	6.2	3.4	7.0	4.6	0.616	0.543		
	Emotional neglect	9.4	4.6	11.0	4.6	14.8	5.4	13.472	**<0.001**	0.10	LF > IF, HF; IF > HF
	Physical neglect	6.2	2.3	6.4	1.9	7.9	3.2	3.219	**0.047**	0.04	LF > HF

a Only effect sizes of significant differences are displayed. Results in bold are statistically significant.

EDE, Eating disorder examination; BDI-II, Beck depression inventory – II; DERS, Difficulties in emotion regulation scale; PID-5, Personality inventory for DSM-5; CTQ-SF, Childhood trauma questionnaire – short form.

### 3.3 Association between group membership and psychopathological variables

Finally, a series of univariate multinomial logistic regressions were run to evaluate the associations between group membership and the variables of interest (Table 4), while controlling for their shared variance. High emotional abuse, low emotion dysregulation, and low emotional neglect were associated with the HF profile of metacognition. Moreover, individuals with greater negative affectivity, emotional neglect, and physical abuse, had higher odds of reporting an IF profile of metacognition. Lastly, for those with high emotion dysregulation and detachment, the odds of reporting an LF profile of metacognition were higher.

**Table 4:** Associations between group membership and psychopathological variables.

		High-functioning	Intermediate-functioning	Low-functioning
		OR (95% CI)	OR (95% CI)	OR (95% CI)
zEDE	Total	0.758 (0.470–1.222)	1.404 (0.892–2.210)	0.810 (0.308–2.128)
zBDI-II	Total	1.227 (0.699–2.151)	0.957 (0.581–1.577)	0.661 (0.226–1.937)
zDERS	Total	0.410 (0.206–0.818)*	1.358 (0.737–2.503)	9.227 (2.022–42.111)**
zPID-5	Negative affectivity	0.518 (0.235–1.142)	2.781 (1.370–5.647)**	0.523 (0.135–2.024)
	Detachment	1.353 (0.690–2.651)	458 (0.251–0.837)*	4.569 (1.348–15.488)*
	Antagonism	1.084 (0.650–1.808)	0.971 (0.634–1.485)	1.238 (0.627–2.444)
	Disinhibition	0.744 (0.357–1.552)	1.192 (0.635–2.236)	0.706 (0.241–2.067)
	Psychoticism	0.653 (0.338–1.261)	1.385 (0.786–2.438)	0.997 (0.407–2.445)
zCTQ	Emotional abuse	2.275 (1.210–4.278)*	0.367 (0.208–0.645)***	1.109 (0.928–1.325)
	Emotional neglect	0.453 (0.245–0.837)*	1.923 (1.104–3.348)*	0.995 (0.839–1.181)
	Physical neglect	1.494 (0.940–2.375)	0.632 (0.407–0.983)*	1.200 (0.879–1.637)
	Physical abuse	0.639 (0.346–1.180)	1.754 (1.030–2.985)*	0.790 (0.550–1.134)
	Sexual abuse	1.185 (0.761–1.846)	0.283 (0.802–0.535)	1.116 (0.925–1.347)
*R*^2^ di Nagelkerke		0.445	0.305	0.491

*p < 0.05, **p < 0.01, ***p < 0.001. Results in bold are statistically significant.

z, Standardized scores; EDE, Eating disorder examination; BDI-II, Beck depression inventory – II; DERS, Difficulties in emotion regulation scale; PID-5, Personality inventory for DSM-5; CTQ-SF, Childhood trauma questionnaire – short form; OR, odds ratio.

## 4 Discussion

The current research aimed to explore different profiles of metacognition across the EDs spectrum using LPA. For this purpose, we conducted a study where various models were assessed using goodness-of-fit statistics, and participant classification accuracy was evaluated using standardized entropy. Subsequently, differences among the derived profiles were examined using Welch ANOVA, followed by *post hoc* tests, and logistic regression models were employed to explore relationships between profile memberships and relevant variables.

A 3-class model emerged for the metacognition domains: “low,” “intermediate,” and “high” functioning. Nearly half of the participants showed high metacognitive functioning, whereas 44.5% of participants showed intermediate metacognitive functioning. Only 7.2% of patients reported low metacognitive functioning. Overall, the present findings support the detection of group heterogeneity in metacognitive functioning among patients with EDs (Rothschild-Yakar et al., 2019; Aloi et al., 2021; Kjaersdam Telléus et al., 2023). Moreover, our findings suggest that the different metacognition profiles are heterogeneous concerning EDs, consistent with previous research (Gagliardini et al., 2020). Specifically, the LF class comprised mostly patients with BN (39%), whereas the IF class included mostly patients with BED (49%). The HF class was the most heterogeneous in terms of diagnostic categories (30% AN-R, 33% BN, and 29% BED). It is worth noting that a trend of increasing severity in all the psychopathological variables (i.e., eating psychopathology, depressive symptoms, emotional dysregulation, dysfunctional personality traits, and CM) was consistently reported across the different profiles of metacognition (LF > IF > HF). Thus, the current findings support the study hypothesis that metacognition may be a transdiagnostic feature across the ED spectrum.

The most striking result of the current study was the association between group membership and psychopathological variables according to the three latent profiles. Interestingly, when partialling out the variance shared by the other CTQ variables, emotional neglect was more strongly associated with the IF profile, while emotional abuse was more strongly associated with the HF profile. There is increasing evidence that childhood emotional abuse is the trauma most associated with disordered eating in adulthood (Michopoulos et al., 2015; Strodl and Wylie, 2020). Our findings add that patients who experienced emotional abuse had higher odds of high metacognitive functioning compared to other groups, when controlling for other forms of abuse and neglect. Therefore, it appears that emotional abuse has the least impact on the metacognitive functioning of individuals with EDs. Further research is necessary to examine whether abusive behaviors, such as constant swearing, yelling, criticism, unrealistic expectations, or unreasonable demands on the child, contribute to the development of mature metacognition among patients with EDs. On the other hand, emotional neglect, characterized by a failure to attend to the child’s emotional needs, may represent a risk factor for developing moderate metacognitive function, especially among patients with BED. Prior research suggested higher levels of emotional neglect than obese patients without BED (Amianto et al., 2018). Thus, further research is needed to examine the link between adverse life experiences during childhood and metacognitive functioning in patients with BED. Interestingly, sexual abuse was not associated with any of the three classes. This result is in contrast with previous literature reporting a high prevalence of sexual abuse among EDs (Afifi et al., 2017) and that a history of sexual abuse could predict poor long-term outcomes (Eielsen et al., 2024). However, the relationship between childhood sexual abuse and eating disorder among adults still requires clarification of the pathways and examination of maladaptive coping responses, with consideration for metacognition patterns. A possible explanation for these mixed findings could be related to the nature of the classes, as the three empirically derived groups were categorized on metacognition features and not according to eating psychopathology. Prior research has also highlighted that the large time gap between childhood sexual trauma and present eating behaviors can convolute total effects (Becker and Grilo, 2011).

Another important finding highlighted in this investigation was the association between detachment PID-5 domain and emotional dysregulation with membership in the LF group. The detachment domain refers to the individual tendency to withdraw emotionally and avoid close interpersonal relations. This result is not surprising, given that few studies have investigated personality traits according to the PID-5 in EDs. Additionally, a recent study found that the facets of anhedonia and depression (traits of the Detachment domain) were strongly associated with all three dimensions of well-being (i.e., emotional, psychological, and social) in patients with EDs (de Vos et al., 2022). In this vein, several studies have demonstrated that patients with EDs reported a pattern of distancing themselves from others, often driven by a desire to protect themselves from potential emotional distress or interpersonal conflicts (Lo Coco et al., 2012; Segura-García et al., 2013; Brugnera et al., 2018; Sivyer et al., 2020; Cassioli et al., 2022; Harris et al., 2023). This interpersonal issue could stem from an impaired metacognition function, specifically the decentration/differentiation subfunction. In fact, this subfunction, which involves the ability to understand others’ thoughts, generate plausible hypotheses about their mental states, and adopt a perspective that is not centered on oneself, is often impaired in several clinical conditions (Semerari et al., 2015; MacBeth et al., 2016; Riccardi et al., 2020; Aloi et al., 2023).

Finally, theoretical frameworks on metacognition have proposed a strong connection between the capacity for metacognitive function and the regulation of emotional states (Euler et al., 2021). Metacognition is considered a crucial process in influencing and adjusting emotional regulation (Rossi et al., 2023), with the roots of this connection believed to be established in early attachment relationships. However, from a psychological perspective, some important differences exist between them. In particular, prior research provided backing for the idea that the capacity for metacognitive functioning might serve as a prerequisite for emotion regulation. This association has been previously linked to increased utilization of adaptive strategies and a reduction in the application of maladaptive emotion regulation strategies (Schwarzer et al., 2021).

### 4.1 Strengths and limitations

The present study is the first attempt to verify whether metacognition, according to the model of Semerari and colleagues (Semerari et al., 2007), can represent a transdiagnostic construct in patients with EDs. It is essential to highlight the inclusion of a large clinical sample comprising both males and females. Notably, the sample exhibits homogeneous frequencies of the main psychopathological diagnoses (i.e., AN, BN, BED), in contrast to previous literature that primarily focuses on female patients with AN and BN. Nonetheless, this work is not without limitations. First, we recognize that this study is exploratory, indicating that certain classes may have a limited number of participants (e.g., the LF group, constituting 7.2% of the total sample). Consequently, it is essential for future studies to cross-validate these findings. However, a rule of thumb is that if the profile includes <1.0% of the total sample size or fewer than 25 cases, the profile should be rejected (Lubke and Neale, 2006; Spurk et al., 2020).

Additionally, this study did not explore diagnoses of personality disorders, which could have offered valuable insights into clinically significant distinctions between the profiles. Hence, the absence of such an investigation may constrain the generalizability of the findings.

Another limitation was the reliance on self-reported data, which may have introduced biases. Furthermore, this research does not include any information about gender identity and sexual orientation, which may hinder the generalizability of the findings to patients with EDs who fall within these sexual minoritized groups. Finally, the cross-sectional design of the study hinders our ability to establish causality and examine the relationships between metacognition and psychopathological variables. With this in mind, further investigation regarding the stability of these latent profiles over time is warranted.

## 5 Conclusion

Present results underscore the significance of considering a metacognitive function in the spectrum of eating disorders. The current data appear helpful both for diagnostic purposes and also for clinicians who deliver psychological interventions. Evaluating metacognition could aid clinicians and researchers in tailoring psychological interventions more precisely. This method allows for active involvement with patients who have a shared mental disorder, but varying levels of metacognitive skills. On the opposite, it also facilitates communication with patients who have different diagnoses, but who share similar challenges in their metacognitive abilities. Our study suggests that patients with EDs can be classified based on impairments in various dimensions of metacognition, regardless of their specific ED diagnosis. Further, it also recommends that clinicians should consider a multidimensional approach to metacognition when treating ED patients. Future studies should investigate whether the three different identified profiles also correspond to different treatment outcomes, given the initial promising results that metacognitive interpersonal therapy seems to show in the treatment of EDs (Fioravanti et al., 2023).

## Data availability statement

Raw data supporting the results of the present study will be made available upon reasonable request from the corresponding author.

## Ethics statement

The studies involving humans were approved by Ethical Committee of “Regione Calabria, Sezione Area Centro” (identifier: Prot. 66/15.03.2018). The studies were conducted in accordance with the local legislation and institutional requirements. Written informed consent for participation in this study was provided by the participants' legal guardians/next of kin.

## Author contributions

MA: Conceptualization, Data curation, Methodology, Writing – original draft, Writing – review & editing. AC: Writing – original draft, Writing – review & editing. GL: Writing – original draft, Writing – review & editing. MR: Data curation, Writing – review & editing. EC: Data curation, Writing – review & editing. RF: Data curation, Writing – review & editing. CS-G: Supervision, Writing – review & editing. ML: Methodology, Supervision, Writing – review & editing.
